# Emergency Department Design in Low- and Middle-Income Settings: Lessons from a University Hospital in Haiti

**DOI:** 10.5334/aogh.2568

**Published:** 2020-01-20

**Authors:** Regan H. Marsh, Kristen D. Chalmers, Keegan A. Checkett, Jim Ansara, Linda Rimpel, Marie Cassandre Edmond, Robert W. Freni, Joshua K. Philbrook, Kimberly Stanford, Shada A. Rouhani

**Affiliations:** 1 Department of Emergency Medicine, Brigham and Women’s Hospital, Boston, Massachusetts, US; 2 Department of Emergency Medicine, Harvard Medical School, Boston, Massachusetts, US; 3 Partners In Health, Boston, Massachusetts, US; 4 Seattle Children’s Research Institute, Seattle, Washington, US; 5 Build Health International, Beverly, Massachusetts, US; 6 Section of Emergency Medicine, The University of Chicago, Chicago, Illinois, US; 7 Hôpital Universitaire de Mirebalais, Mirebalais, HT; 8 Zanmi Lasante, Croix-des-Bouquets, HT; 9 ADAPTIV Architecture and Planning, Beverly, Massachusetts, US; 10 DENS Facility Services, Somerville, Massachusetts, US

## Abstract

**Background:**

Studies from high-income settings have demonstrated that emergency department (ED) design is closely related to operational success; however, no standards exist for ED design in low- and middle-income countries (LMICs).

**Objective:**

We present ED design recommendations for LMICs based on our experience designing and operating the ED at Hôpital Universitaire de Mirebalais (HUM), an academic hospital in central Haiti. We also propose an ideal prototype for similar settings based on these recommendations.

**Methods:**

As part of a quality improvement project to redesign the HUM ED, we collected feedback on the current design from key stakeholders to identify design features impacting quality and efficiency of care. Feedback was reviewed by the clinical and design teams and consensus reached on key lessons learned, from which the prototype was designed.

**Findings and conclusions:**

ED design in LMICs must balance construction costs, sustainability in the local context, and the impact of physical infrastructure on care delivery. From our analysis, we propose seven key recommendations: 1) Design the “front end” of the ED with waiting areas that meet the needs of LMICs and dedicated space for triage to strengthen care delivery and patient safety. 2) Determine ED size and bed capacity with an understanding of the local health system and disease burden, and ensure line-of-sight visibility for ill patients, given limited monitoring equipment. 3) Accommodate for limited supply chains by building storage spaces that can manage large volumes of supplies. 4) Prioritize a maintainable system that can provide reliable oxygen. 5) Ensure infection prevention and control, including isolation rooms, by utilizing simple and affordable ventilation systems. 6) Give consideration to security, privacy, and well-being of patients, families, and staff. 7) Site the ED strategically within the hospital. Our prototype incorporates these features and may serve as a model for other EDs in LMICs.

## Background

Emergency care (EC) can improve health outcomes and reduce disparities [1]. Despite this, EC is often extremely limited in low- and middle-income countries (LMICs), including Haiti [2,3]. In addition to investments in education, human resources and operations, developing successful emergency care systems requires designing, building and maintaining high-quality emergency departments (EDs).

Studies from high-income settings have demonstrated that an ED’s physical infrastructure is closely related to its operational success [4,5,6]. However, no standards exist for ED design in LMICs. Uniform application of design principles from high-income settings would be inappropriate due to variations in disease burden, staff training, health system characteristics, and financing. To address this gap, we present our experience with ED design at Hôpital Universitaire de Mirebalais (HUM), an academic hospital in central Haiti.

As part of a quality improvement project to redesign the HUM ED, we collected feedback on the current design from key stakeholders to identify priority design features impacting quality and efficiency of care in our setting. We present key lessons learned and recommendations for ED design in LMICs, and offer our proposed redesign for the HUM ED as a model.

## Local context

Founded in Haiti in 1983, the nongovernmental organization Partners In Health (PIH) has worked in partnership with the Ministry of Health to provide health care in the impoverished rural central areas of the country. Medical care in Haiti is affected by limited physical infrastructure. In response to the earthquake, the Ministry of Health requested that PIH construct an academic teaching hospital to improve clinical service delivery and medical education training programs.

Opened in 2013 and operated in partnership between the Ministry of Health and PIH, HUM is a 300-bed public referral hospital and academic medical center located 1.5 hours from Port-au-Prince, offering outpatient and inpatient services for medicine, pediatrics, OB/GYN, surgery, intensive care, neonatology and emergency care. It is one of four academic hospitals in Haiti and hosts the country’s only emergency medicine (EM) residency program. From the beginning, emergency care was identified as a priority. In 2014, the EM residency program opened. Currently, the department and the program are entirely locally run.

**Figure 1:**
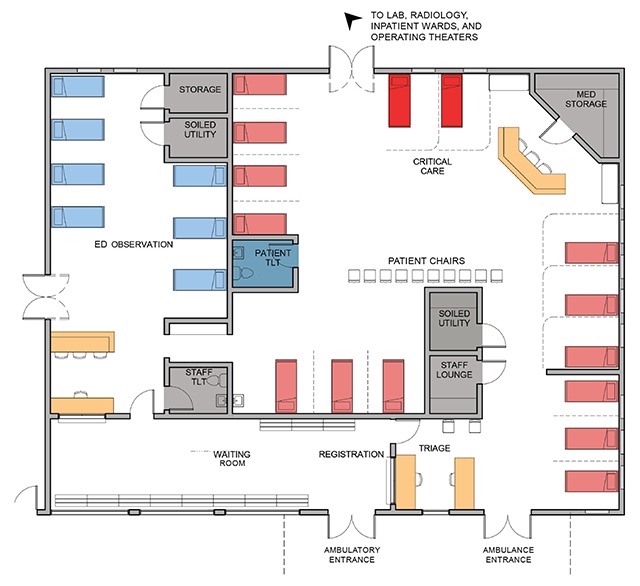
The existing HUM ED. The original ED featured only 15 beds (pink and red above). To expand capacity an observation area with seven beds (blue) and central chairs where patients can be seen were added.

The HUM ED has over 14,000 patient visits annually, of which approximately 25% is pediatrics and 20% trauma. The ED was designed and built as a 4,500 square-foot, 15-bed unit. Since opening, design modifications have been implemented in response clinical and operational needs, including adding a 7-bed observation area and chairs for patient overflow (Figure 1). Even with these additions, the ED faced increasing patient needs. Hospital and ED leadership, as well as clinical staff, recognized that an expansion was necessary and advocated for a redesign to improve quality of care.

## Methods

As part of a quality improvement project, semi-structured interviews—focusing on design features that promote high quality and efficient care—were conducted with key stakeholders. These included HUM’s EM leadership, attending physicians, residents and nurses, as well as hospital administrators, facilities managers and hospital design architects. Interview responses were combined with operational experience and feedback over time at department staff meetings. This feedback was collated and reviewed for key themes. Based on this stakeholder feedback and our operational experience, we offer key lessons learned in seven domains of ED design in LMICs.

## Design Recommendations

### 1. The “Front End”: Triage and Waiting Areas

Triage ensures timely evaluation of critical patients and is essential to emergency care, but remains underdeveloped in LMICs. A thoughtfully-designed triage space facilitates triage’s successful implementation, reduces wait times for ill patients, and ensures the safety of those waiting to be seen [7]. We suggest a dedicated triage area adjacent to registration, between the waiting room and main department, with clear, one-way flow, ensuring that every patient is triaged on arrival. Triage should have easy access to the main treatment and resuscitation areas for patients needing immediate stabilization, and visibility into the waiting and arrival areas, so staff can monitor waiting patients for changes in condition.

The waiting area must meet the specific needs of LMICs where patients’ family members play an essential role in their care. Many travel long distances and stay at the hospital overnight. In our current ED, waiting families mix with patients waiting to be seen, making it hard to detect clinical changes in the patients. We recommend separate waiting areas to facilitate patient monitoring while providing dignified rest space and bathrooms for accompanying family.

### 2. ED Size and Physical Layout of the Interior

#### ED Size

Patient volume in the HUM ED has consistently exceeded the designed capacity, due to both higher than anticipated demand for care and long length of stay (LOS). We are currently required to treat 10–25+ patients in central chairs, where privacy and care are constrained. When planning ED size, we recommend evaluating local factors that influence patient throughput and therefore patient census (Table 1), rather than extrapolating from calculations developed for high-income settings. While building additional space is not a solution for inefficiencies that delay throughput, a realistic assessment ensures ED size is functional.

**Table 1:** Factors to consider when planning ED size. ED patient census is affected by ED throughput (internal factors), determinants that impact arrivals (input factors) and those that impact disposition (output factors). For example, in settings where patient volumes markedly fluctuate by season or time of day, it may be necessary to have areas of the ED that can open and close as needed.

Input factors	Internal Factors	Output Factors

**Demand** Availability of other EDs Patient fees at others EDs Perturbations in the healthcare system such as strikes limiting access to other facilities **Variable Patient Volumes** Reduced patient arrival at night due to limited transportation Seasonal variation in disease burden Mass casualty incidents (MCIs)	**Staff** Training and capacity Staff to patient ratios **Patients** Average patient complexity and acuity Delayed presentations compared to high-income settings **System** Extended wait times for radiology and laboratory tests Limited access to specialty consultation	**Admitting capacity** Hospital crowding Hospital policies to manage throughput **Discharge capacity** Few skilled nursing facilities and rehab hospitals as alternatives to admission Structural factors: poverty and limited water and sanitation limit home care and impact safe discharge Large catchment areas and transport costs make return visits for follow-up difficult Limited nighttime transportation may prevent evening discharges

#### Physical layout

Ensuring line of site from staff to patients is a primary ED layout consideration that influences patient care [5]. Visual monitoring to detect changes in clinical condition is essential in LMICs, where staff are often fewer and electronic monitors rare. We recommend an open-style ED, with a designated area for critical patients near a central nursing station. Defining this resuscitation space indicates to staff which patients need close monitoring and frequent or advanced interventions, and allows critical equipment to be clustered.

Flexibility in space is key, as LMIC EDs face both predictable and unexpected variations in demand. For example, EC demand at HUM increased when the 2016 Haitian public hospital strikes restricted access to other facilities [8]. To create flexibility, balance multi-purpose spaces with discrete areas for different patient acuities. Consider spaces that can close overnight, such as a fast track area for low acuity patients. Designate overflow areas for mass casualty incidents where many patients arrive at once. In the HUM ED, we use a wide hallway adjacent to the ED for this purpose.

### 3. Organization of Materials and Supplies

We recommend building storage spaces sufficient to manage large volumes of supplies, as limited supply chains can make frequent ED resupply difficult necessitating storage of larger supply quantities. Supplies should include the essential medications and consumable materials necessary for EC. A hospital-wide backup supply room can also ensure 24/7 access to critical supplies and medications in the event of ED stockouts.

In the HUM ED, we use and recommend carts that can roll to the bedside for frequently used supplies (IV placement, resuscitation equipment, orthopedic interventions, suturing, etc.), as at-bedside storage is typically not feasible.

### 4. Oxygen and Suction

We strongly recommend investing in reliable O_2_. Simply put, oxygen saves lives. Each system has advantages and disadvantages (Table 2). At HUM, we opted for a central concentrator that distributes high-flow O_2_ throughout the hospital via bedside wall-accessed ports. For many EDs, we recommend a piped-local system from an ED-specific manifold of cylinders to maximize benefits relative to cost. Regardless of the option chosen, ensure a plan for ongoing maintenance and operations.

**Table 2:** Overview of the advantages and limitations of oxygen systems. Options with wall-access are clinically convenient, but require more maintenance, while any choice involving O_2_ cylinders is laborious and requires mechanisms to refill and replace cylinders. Cylinders may run out without being noticed and may fall over.

Oxygen System	Wall-accessed	High-flow O_2_ (15 L/min)	Requires O_2_ Cylinders	Requires electricity	Requires space at bedside	Capital cost	Operational cost	Maintenance effort	Overall Recommendation

Piped from centralized O_2_ concentrator	+	+	–	+	–	$$$$	$$$$	++++	****
Piped from a local manifold of O_2_ cylinders	+	+	+	+	–	$$$	$$	++	***
Individual bedside concentrators	–	–	–	+	+	$$	$$	++	**
Bedside cylinders	–	+	+	–	+	$	$$	+	*

Similarly, HUM utilizes a centralized hospital-wide suction system. Like our central O_2_ system, it is clinically convenient but has high upfront costs and ongoing maintenance requirements. Given the lower clinical demand for suction compared to O_2_, based on our experience, we recommend considering lower-cost alternatives, including smaller ward-specific wall suction systems or portable bedside suction units.

For both oxygen and suction, we advise prioritizing system-redundancy. For centralized systems, this includes maximizing access points (wall outlets), to accommodate fluctuations in patient volumes. For any oxygen system, ensure backups and small cylinders for patient transport.

### 5. Infection Control and Ventilation

Poorly ventilated and overheated spaces lead to worker fatigue, patient discomfort, infection risk, improper medication storage and biomedical equipment malfunction [9,10,11]. Expensive ventilation systems may be cost-prohibitive, and well-designed, naturally-ventilated spaces can have higher rates of air exchange and lower infection risk than mechanically-ventilated rooms [12].

Temperature and ventilation were frequent critiques of the HUM ED, where nearby surrounding buildings limited the efficacy of the original passive ventilation design. Through our team’s subsequent experience at multiple hospitals in LICs, we developed a four-tiered approach to ventilation (Table 3). We retrofitted the HUM ED with a relatively inexpensive mechanical system to augment airflow, with some improvement in the ED’s climate (option 3). For most EDs, we recommend an improved passive ventilation scheme (option 2) that takes advantage of natural thermodynamics and balances cost with climate control.

**Table 3:** Overview of construction and operating costs of different ventilation options. Energy costs are based on HUM ED size and electricity costs in Haiti.

Ventilation Strategy Options	Description	Construction*	Annual Energy + Maintenance	Projected 10-year cost

**1) Passive ventilation**	Flat roof Air flows from low-height intakes to elevated louvers	$0	$0	$0
**2) Improved passive ventilation with elevated roof**	Elevated, vented roof allows hot air to exit Cooler air flows from low-height intakes, up to sloped roof 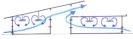	$32,000	$0	$32,000
**3) Mechanical Ventilation**	Flat roof Air is forced through mechanical whirly birds 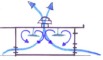	$8,500	$3,750	$46,000
**4) Air Conditioning**	Flat roof Climate control achieved through ED-wide air conditioning	$25,000	$12,000	$145,000

* Costs above baseline of a traditional passive ventilation scheme for an ED the size of HUM based on construction costs in Haiti.

**Figure 2:**
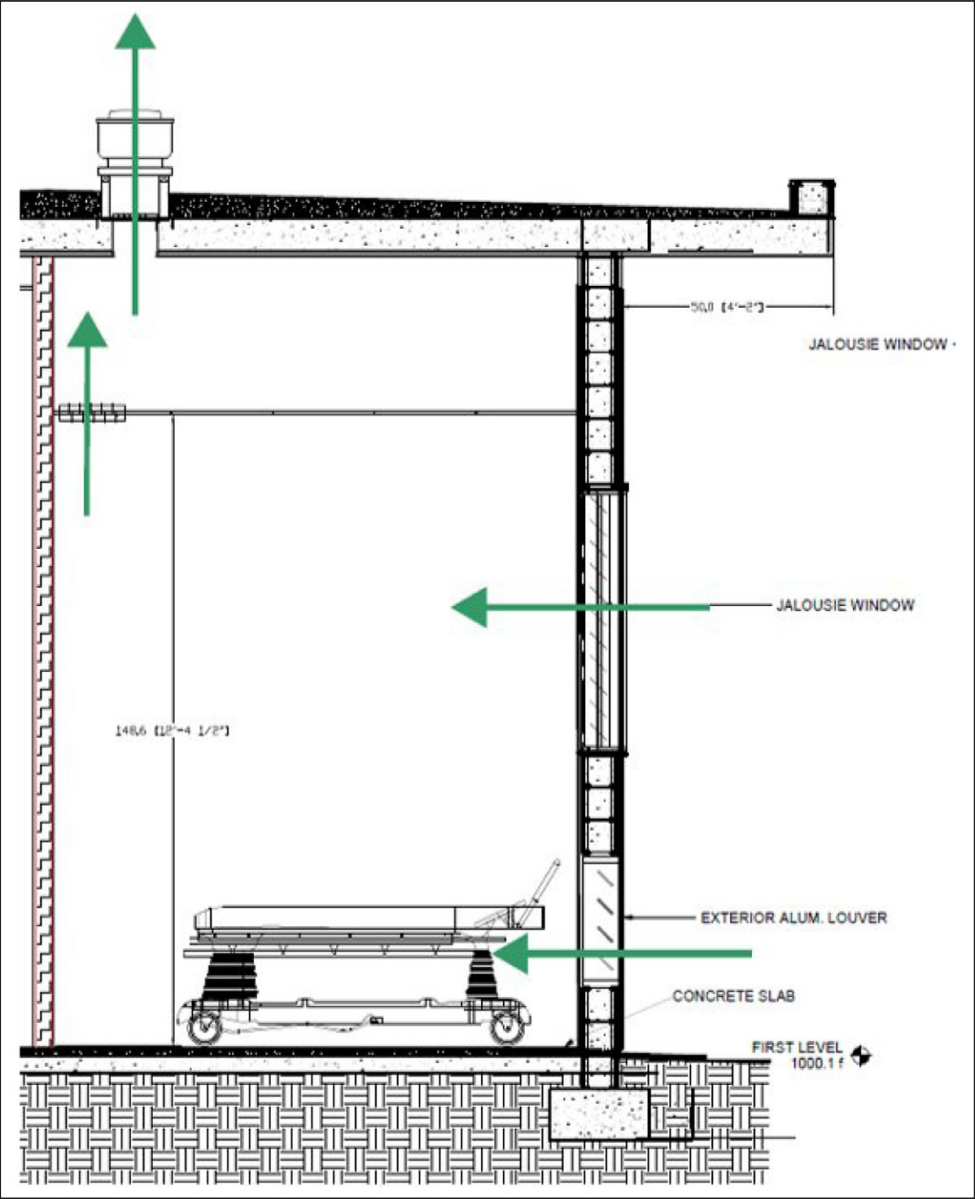
Negative pressure is achieved in an isolation room using mechanical ventilation and unidirectional airflow.

Ventilation schemes can also create isolation capacity for airborne pathogens, including tuberculosis. Isolation rooms have higher cost-per-bed than general ED beds, but they can be affordably constructed utilizing a mechanical ventilation system to create negative pressure (Figure 2).

From our experience in Haiti, this system costs less than $1,200 per room to install, uses less than $50 worth of energy annually and meets the established standard of 12 air-changes per hour [9]. The appropriate isolation capacity should be determined according to local burden of disease and availability of other designated treatment facilities (tuberculosis, cholera, Ebola, etc). Patient monitoring in airborne isolation rooms can be ensured with strategically-placed windows.

Several other easy design choices can improve infection control. Throughout the ED, well-positioned sinks can promote handwashing. Additionally, adequate bed spacing mitigates the spread of infection [13] and improves care delivery. We have found one meter between beds reasonably balances cost, clinical care and infection control needs. Upper-room ultraviolet lights provide effective, low-cost, germicidal irradiation to reduce transmission of airborne diseases [14]. Lastly, throughout the ED, choose easily sanitized finishings; we recommend sealed floors (not tile), non-cloth furniture, and removable, easy-to-clean curtains or patient dividers.

### 6. Security and Well-being

Staff and patient safety and well-being are essential to delivering quality emergency care. EDs face unique security risks, including increased risk of violence against healthcare workers [15]. In any design, local context should inform the necessary level of security, and ED accessibility and routes of egress in an emergency should be balanced with controlled access. The initial HUM design prioritized accessibility but had multiple unsecured access points. Since opening, we have enhanced security with select locked doors, one-way glass, and security personnel. Adding one-way film to safety glass on exterior doors can affordably enhance ED privacy and security without sacrificing visibility into the waiting room or exterior spaces; this is approximately $225/window versus $940/window for one-way glass itself. As needed, consider designs that allow for ED lockdown with safe spaces for staff in high-risk situations.

Often overlooked, privacy is paramount to patients and a priority for quality care delivery. Privacy can be enhanced in an open ward by including limited private exam rooms in addition to curtains and screens. To promote dignity, sufficient bathroom facilities and dedicated space for waiting families should be included.

Staff wellness can be promoted through staff restrooms, break rooms, and secure storage for personal belongings. Additionally, dedicated workspace for doctors and nurses within the main ED facilitates documentation and allows staff to perform their activities in comfort without compromising patient monitoring. Lastly, planning purposeful space for a patient tracking white board improves ED management and offers space for on-shift education.

### 7. Adjacencies

Lastly, the location of the ED is key to its function both within the hospital and the surrounding community. The HUM ED is easily visible from the road but is clearly separated from the main entrance. By design, the ED is close to radiology and lab services, operating theatres and the intensive care unit. We recommend these adjacencies given the importance of these clinical relationships. Additionally, to facilitate patient flow, we recommend covered pathways large enough to accommodate a stretcher between the ED and other wards, radiology, the kitchen, laundry, and morgue.

## Discussion

As efforts to strengthen emergency care in LMICs continue, purpose-built EDs will be key to improving care quality, patient outcomes, and staff safety and well-being. Foresight and consideration in these seven domains will maximize utility within a given construction budget and position EDs for operational and maintenance success.

**Figure 3:**
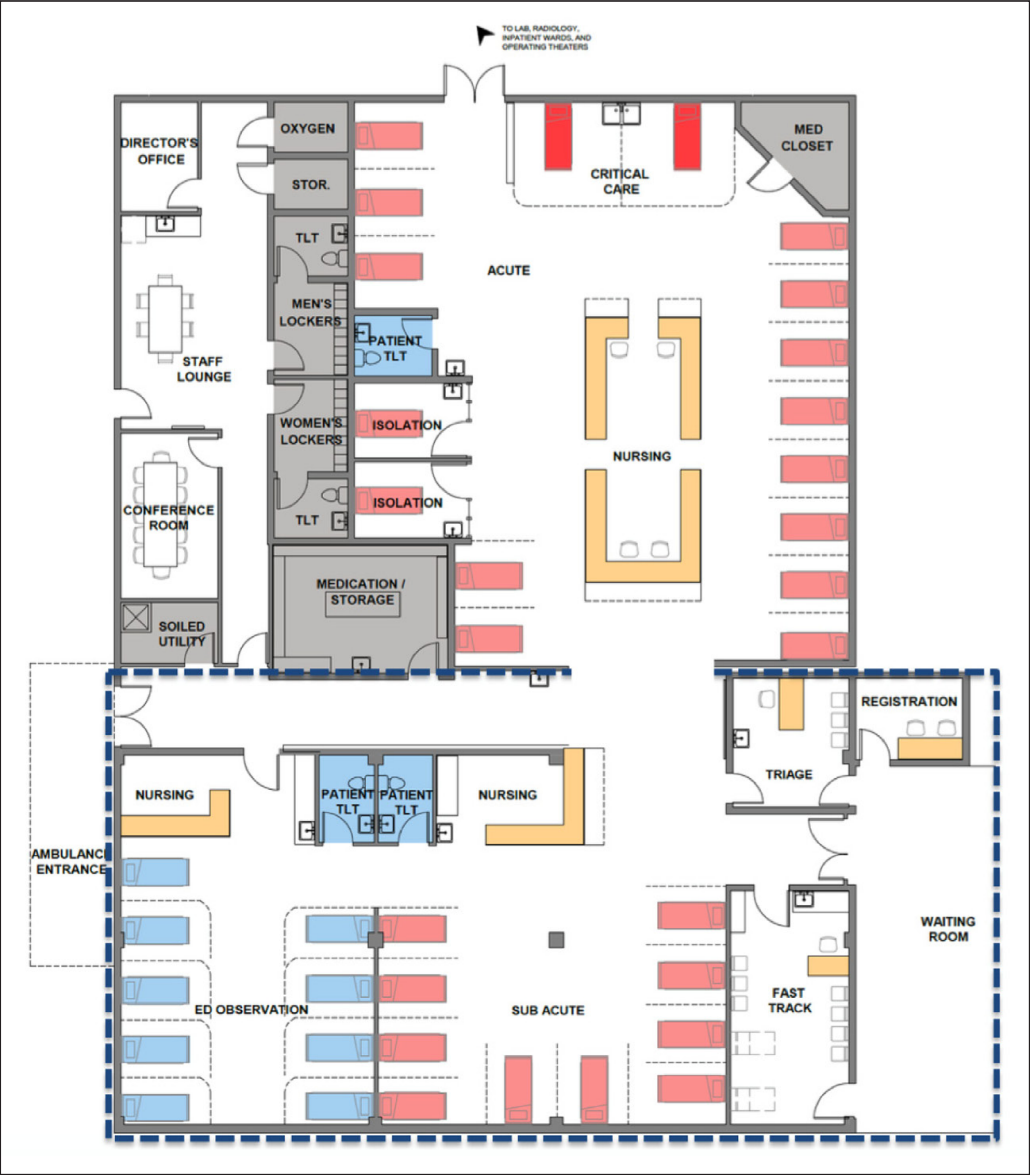
HUM ED Redesign. The dashed box indicates new construction, whereas the remainder of the space reflects redesign within the existing footprint of the HUM ED. Patients move from the waiting area to dedicated triage space into the appropriate fast-track, acute, sub-acute, or observation areas. Staff workspace is positioned to prioritize line-of-site to critical patients.

Our newly designed HUM ED (Figure 3) incorporates improvements in seven design domains. The new design is larger given the high demand for care and relatively long LOS. Designated subacute and fast track areas promote flexibility to accommodate fluctuations in patient volume. There is an expanded triage area, incorporating triage into patient flow, and improved lines of sight within the ED to all patients. The waiting area can be separated for patient waiting and family waiting. We will continue to supply oxygen through a piped system but will add additional ports. Since a pop-up roof is difficult to retrofit, we will ameliorate climate control with air conditioning. Infection control will be strengthened with added sinks and mechanically-ventilated negative pressure isolation rooms. The wellbeing and dignity of patients, family, and staff will be enhanced with expanded bathroom facilities, upgraded security with limited access points, and a staff lounge. The new ED continues to take advantage of our current adjacencies within the hospital.

These recommendations reflect our best current understanding to immediately improve infrastructure for ED care within health systems in LMICs. Though some recommendations may differ from those in high-resource settings, we believe these represent an important step in providing high quality and equitable emergency care. As EM develops and health systems improve, these recommendations will require adaptation.

A number of unanswered questions regarding ED design in LMICs remain. Future research should quantify the impact of different ward design options on quality of care, safety and monitoring, and patient privacy and satisfaction in LMICs. Additional studies could consider the best mechanisms for balancing security with accessibility, and future work should include a formal costing analysis of ED design and construction, which will vary by local setting.

Our findings should be considered relative to several limitations. First, our recommendations are drawn from our collaborative experience in a relatively large referral hospital in Haiti. However, our team has implemented aspects of these recommendations in other contexts (including smaller hospitals) with positive outcomes, and we anticipate many recommendations are generalizable. Second, our new prototype is slated for construction but not yet in use, so additional considerations could emerge. Third, while we solicited broad input, this represents a quality improvement project rather than a research investigation, so there may be viewpoints not reflected here.

As emergency care continues to expand, purposeful ED design can improve care delivery, quality, and efficiency. We hope that these recommendations provide a foundation for ED infrastructure in other LMICs to improve clinical care delivery and patient outcomes.
